# Priority of Vaccination of the Population against COVID-19: Moral Principles

**DOI:** 10.3390/vaccines11020380

**Published:** 2023-02-07

**Authors:** Tsuriel Rashi

**Affiliations:** School of Communication, Ariel University, Ariel 40700, Israel; tsuriel.rashi@gmail.com

**Keywords:** Priority of Vaccination, COVID-19, Morality

## Abstract

The COVID-19 pandemic broke out at the end of 2019 and throughout 2020 there were intensive international efforts to find a vaccine for the disease, which has already led to the deaths of over 6 million people. In December 2020, several pharmaceutical companies announced that they had succeeded in producing an effective vaccine and after approval by the various regulatory bodies, countries started to vaccinate their citizens. With the start of the global campaign to vaccinate the world’s population against COVID-19, there was a strong renewal of the debate about prioritizing the population for the vaccination. This article presents the moral approaches to this issue and their consequences.

## 1. COVID-19 and the Global Efforts to Vaccinate

Vaccinating a whole population, which is a preventive medical service that a country owes its citizens, is based on the recommendations of professionals and health authorities around the world, which regularly arrange for the inoculation of the residents of their countries against dangerous infectious diseases. Vaccination has proved to be one of the last century’s most important advances in public health [1], and the basket of vaccinations is updated from time to time according to need. The medical community considers it one of its major achievements, as it has prevented the spread of deadly diseases in an effective and efficient manner. 

The COVID-19 pandemic has affected hundreds of millions of people and has had fatal consequences for more than 6 million. The pandemic apparently erupted in December 2019 in the city of Wuhan, China, and by the middle of February 2020 had started to spread, causing panic, which was soon followed by economic crises around the world. In December 2020, the Moderna, Pfizer, and AstraZeneca drug companies developed successful vaccines against COVID-19 and received approvals from various regulators, including the FDA in the United States and the European EMA. 

As many countries around the world embarked on a mass vaccination effort, the question arose as to which segments of the population should be inoculated first. In principle, there was general agreement that medical teams should be the first to receive the vaccine because they are the “combat soldiers” on the “front line” of the war against the virus. However, there is an ongoing discussion about the nature of the second tier and subsequent recipients; whether the deciding factor should be their degree of risk owing to their age and health status or the extent of exposure to the general public, such as teachers and educational staff, or perhaps their relative importance in public life, such as government ministers and senior officials.

A second option is not to prioritize according to clear groups in the population but to give the first dose of the vaccine to the whole population, as some countries have already decided, and some that will soon make that decision. According to that protocol, the second dose would be given to the entire population in the distant future. The medical and moral justification was that it is right to give the entire population at least partial protection than to fully protect certain groups. These countries—such as the United Kingdom—made this decision despite a report published in the *New England Journal of Medicine* [2]. Reports from Israel, which was at the top of its population’s vaccine table, showed that a single dose of the Pfizer vaccine might be less effective than expected [3]. 

This issue of prioritization of vaccination was extremely critical because vaccination has become a medical, social, legal, moral and religious obligation in many countries around the world [4] (The different religions also dealt with the appropriate altruistic behavior during the epidemic and the various measures to reduce the morbidity [5]). This obligation means that it is very difficult to continue the routine of life without the vaccine against COVID-19 on the one hand, and the creation of the high–and perhaps even complete–dependence on the manufacturers of the vaccine [6]. The pages that follow deal with the moral approaches regarding these medical dilemmas.

## 2. The Various Approaches to Prioritizing Vaccines—Comparative View

The US National Academy of Medicine (NAM) and the World Health Organization (WHO) proposed a framework for COVID-19 vaccine allocation [7]. Health professionals in, for example, Indonesia argue that younger people should be vaccinated first, the rationale being that younger working adults are generally more active and more social, so this strategy would be the fastest way of decreasing community transmission [8]. According to Emanuel et al. [9], benefiting people and limiting harm is a universal value, so they proposed the Fair Priority Model to ensure fair distribution of vaccine among countries. They focused on three types of harm caused by COVID-19. First, it kills people and causes permanent organ damage. Second, it indirectly harms health even for the uninfected by straining health care systems, thus raising mortality rates for common conditions, and accelerating the spread of disease by interfering with regular immunizations. Third, it has devastated the global economy. Economics and health interact: worsening economic conditions harm health and a worsening pandemic harms the economy; thus, allocators must decide where a vaccine’s harm-reducing powers are most urgently needed. 

NAMs proposal suggests another value: reciprocity. That is, recognition of past actions such as risking oneself to benefit others. Vaccine distribution should be focused on current and future benefits for vaccinated individuals, on protecting individuals from the spread of infection, and on the socioeconomic benefits that accrue as the health system and societal functioning improve. 

Emanuel et al. [9] also contends that countries should show preference to their own citizens over those of other countries (an example of such a claim arose against Israel, which receives a regular supply of Pfizer vaccines in exchange for sharing the accumulated medical information with the pharmaceutical company. In the meantime, Israel holds about 100,000 doses made by a Moderna company and is not ready to distribute these vaccine doses as of the beginning of February 2021. For more information on this matter, see Levingston [10]) only until the pandemic starts to show signs of a decline [9].

According to Bubar et al. [11], governments should follow a model-informed approach to vaccine prioritization that evaluates the impact of prioritization strategies on cumulative incidence and mortality. Owing to the fear that there will not be enough resources to keep up with the global and national rate of infection, various countries have begun to write protocols for prioritizing the distribution of medical resources. For example, following the outbreak of the current pandemic, the director general of the Israeli Ministry of Health appointed a committee to formulate rules for prioritizing the treatment of COVID-19 patients (The Committee was made up of specialists from the areas of health, ethics, law, society, and Jewish, Christian, and Muslim law. Rabbi Prof. Abraham Steinberg, Israel Prize laureate for Torah literature, a physician and an ethics expert who served as joint chairperson of the committee). Israel was the first country in the world to receive the vaccines from Pfizer, and which should have been the first in the world to deal with the prioritization of the distribution of vaccines among the medical teams and all citizens. In this respect, Israel was different from all the countries of the world. The committee opened its recommendations with the following declaration:

The fundamental starting point for patients in need of medical resources was the value of life as a supreme value and the basic equality of all people. “Thus man was created alone, to teach you that whoever loses a single soul is as though he lost an entire world, and everyone who saves one life is as though they have maintained the whole world” [12]. The equality required for ethical and legal reasons determines that one cannot discriminate among patients on grounds of religion, race, sex, nationality, country of origin, sexual orientation, socio-economic situation, social status, marital status, civil status in the country, occupation, age, and more.

The Committee emphasized that “the sole deviation from the principle of equality is only on the basis of medical differences in the potential success of the treatment and the chances of survival”:

The medical team will not receive priority unless the matter is necessary in order to overcome a shortage of staff, whether returning to work after they had recovered or as an incentive for volunteering. Where there is an equality in medical status between two patients, the fact that one is a member of the medical team will be deemed a preferential point.

How does one set medical treatment priorities for patients whose chances of recovery are equal? Subcommittees of the Joint Committee were divided on this issue. Some members of the Legal Subcommittee favored casting lots, “because this random tool is more moral and ethical for such a difficult decision,” whereas others believe that “we should leave the decision in the hands of the doctors, while explicitly stating that this was in extreme circumstances and without any decisive difference among those waiting.” In contrast, members of the Medical Subcommittee contended that “one should use the ‘first come, first served’ principle... and not an allocation based on a random casting of lots that is alien to medical practitioners and which will lose valuable time.” The latter’s position was the one adopted by the Joint Committee [13].

The common denominator among all these research and regulatory bodies as well as the scientists mentioned above is that they addressed providing full and comprehensive care to the person who comes for treatment or vaccination. In other words, they were not dealing with a situation in which it was possible to give partial, equal treatment to the entire population rather than providing full treatment to particular segments. That dilemma arose when various countries had to make decisions regarding their policies for vaccinating their populations against COVID-19.

## 3. The Ethics of Distribution of Limited Resources—Between Rome and Jerusalem

How should a country prioritize vaccine distribution when inoculation with many COVID-19 vaccines requires two doses in order to achieve maximum efficacy. Should the entire population be given one dose so as to provide partial protection against COVID-19 or should two doses be administered to certain groups? Pharmaceutical companies Pfizer and Moderna have explicitly stated that for the best effect the vaccine must be given in two doses 21 days apart. However, as the rate of production does not meet global requirements, as noted above, some countries have decided to give only the first dose to their entire populations to be followed up by the second dose sometime in the distant future. The medical and moral justification for this decision is that it is better to give the entire population at least partial protection than to fully protect certain groups. The outstanding example of this approach is the British policy, where the model for distribution has engendered a widespread debate both within and outside the United Kingdom. 

The UKs healthcare regulator, the Medicines and Healthcare Products Regulatory Agency (MHRA), approved the world’s first COVID-19 vaccine for emergency use in December 2020. Less than a month later, it approved AstraZeneca (AZ) and the University of Oxford’s adenovirus candidate. In the face of an escalating pandemic in the United Kingdom, the MHRA recommended spacing out two doses of the vaccines by up to twelve weeks to optimize the UKs inoculation program. and also called for rescheduling of existing appointments for the second dose of the Pfizer/BioNTech vaccine. 

There were some concerns in the scientific community about the MHRAs decision to change the scheduling protocol, owing to the question of whether extending the time between doses, particularly for the Pfizer/BioNTech vaccine, was in fact the best possible option. In an attempt to allay some of these concerns, one day after MHRA announced its decision, the Joint Committee on Vaccination and Immunisation (JCVI) published its reasoning for advising the dosage gap extension. It explained that “rapid delivery of vaccines is required to protect those most vulnerable [14], and that models suggest that initially vaccinating a greater number of people with a single dose will prevent more deaths and hospitalisations than vaccinating a smaller number of people with two doses” [15].

The dilemma of the distribution of limited resources appears in the world’s philosophy literature in both Greco-Roman and Jewish thought. At this time, it receives added meaning in the light of the complex situation that has been created. In the Babylonian Talmud, we read about a disagreement between two first-century Sages, Rabbi Akiva and Ben Petura.

The two men are on a journey, and one has a canteen of water: if they both drink, they will both die, and if one of them drinks, he will reach a town. Ben Petura said that it was better if both drank and then died, so one should not see the death of his friend. Until came Rabbi Akiva and taught: “Let your brother live with you” [16]–your life takes precedence over the life of your friend [12].

Among the many books and articles that refer to the story found in Baba Metzi’a in the context of a general reflection about Jewish ethics, without mentioning the Greek and Latin sources in any way [17,18,19].

According to Berthelot [20], early philosophical and the rabbinic texts deal with similar questions, which indicates that the Jewish Sages were aware of a range of ethical debates in the Greco-Roman world. 

Little of the Stoic literature from before the turn of the common era is extant, Thus, to read the Stoic philosopher Hecato, a disciple of Panaetius, we have to rely on the writings of the Roman statesman, lawyer, and scholar Marcus Tullius Cicero (106–143 BCE), who was not himself a Stoic but knew Stoicism well. In *De officiis*, he wrote [21]:

The sixth book of Hecato’s work On Obligations is full of questions like these: is it right for a good man to fail to feed his retinue of slaves when the price of corn goes sky-high? Hecato presents the arguments on both sides, but he concludes by aligning our obligation with what he regards as the useful rather than with humane conduct. Again he asks: if a loss had to be sustained at sea, would it be preferable to lose an expensive horse or a lowly slave? In this case the owner is drawn one way by family property and another by feelings of humanity.

If a fool lays hold of a plank in a shipwreck, should a wise man grab it from him if he can? Hecato says no, on the grounds that it would be unjust. “Very well, but should a ship’s owner seize the plank that is rightfully his?” “No, he has no more right to do that than to throw a passenger overboard in mid-ocean merely because he owns the ship: for until the ship reaches the destination for which it was chartered, it belongs to the passengers, and not to the owner.”

Another question: assuming that there is one plank and two shipwrecked passengers, both of them wise men, should each try to grab it for himself or should one yield to the other? “One should give way, yielding to the one whose life is more important whether intrinsically or to the state (*Cedat vero, sed ei, cuius magis intersit vel sua vel rei publicae causa vivere*).” But supposing the balance is equal on both sides? “Then there will be no contest. One will yield to the other as if in a lottery or a game of chance.”

It is appropriate to compare this solution to one proposed by the Islamic philosopher and sage Abu-Bakr Al-Razi, who discussed a case similar to one about which Rabbi Akiva and Ben Petura disagreed. Familiar with the story of the people wandering in the desert from a lost Greco-Roman source, Al-Razi offered a solution that has a striking resemblance to the one attributed to Hecato, noting that the water should be given to the person who is most useful to humankind [22]. 

Cicero himself also raised the question of the proper conduct between two people in relation to limited resources:

If there is a shipwreck, and two men are clutching a plank which can support only one, what is the stronger of the two to do? If he lets go, he will be a just man and he will drown! If he is wise, he will be unjust and send the other man to his death [23].

The Roman Stoic philosopher and statesman Lucius Annaeus Seneca (4 BCE–65 CE), also dealt with the issue and in his book *On Benefits*, we read:

Let us never bestow benefits that can redound to our shame. Since the sum total of friendship consists in putting a friend on an equality with ourselves, consideration must be given at the same time to the interests of both. I shall give to him if he is in need, yet not to the extent of bringing need upon myself; I shall come to his aid if he is at the point of ruin, yet not to the extent of bringing ruin upon myself, unless by so doing I shall purchase the safety of a great man or a great cause [24].

In this case, all “schools” basically agree that to seize the plank or the bottle from one’s companion by force is an injustice. Only the pragmatic Cicero added that when the interest of the state or society is at stake, appropriation of someone else’s goods is permissible, even if it potentially deprives him of his means of survival. However, the rabbinic texts never considered that a legitimate choice. (For a comprehensive discussion comparing the different versions of the nature of the dilemma and the different ways to solve it as presented by the Jewish and the Greco-Roman worlds, see: Kaminka [25]; Pines [26]; Lieberman [27]; Halevy [28]; Urbach [29]. For a summary of the conclusions reached by these scholars, see Berthelot [20]. For a comparison between the Jewish and Christian worldviews around the issue of self-sacrifice, see Litwa [30]). Further, the Talmud [12] declares that no individual is entitled to decide that his life is worth more than the life of his fellow: “What reason do you see for thinking that your blood is redder? Perhaps his blood is redder” [20].

Thus, it can be said that according to Rabbi Akiva, just as I have no right to destroy the life of someone else to save my own life, so I do not have the right to destroy my own life for that of someone else, since we are both people, and our lives have the same value. Rabbi Akiva established a moral principle: one own’s life takes precedence over the life of another. In contrast, Ben Petura was motivated by feelings of human brotherhood and mercy, even if as a result two people die. He thought that there are more important values than the sanctity of life, such as friendship, kindness, and mercy. He considered that life after watching your friend die is worse than death and that all life after that is no longer worthwhile. It can be said that the greatness and humanity of a man who overcomes his wish for life in order to grant life to someone else are what separates us from animals. The ability to master the self is what elevates man’s spirit and gives life reason and meaning. Ben Petura contended that it is not right that one should live and the other die, so it is better that they both die.

The foregoing discussion clearly raises a difficult potentially broad ethical issue that has many implications for various global societal relationships (An example of the debate regarding this issue can be seen in Paterson [31]). One consequence of the current reality regarding distribution of COVID-19 vaccines could well be that a particular country that has an inventory of vaccines is asked to share its supply with another country so that it can also vaccinate its citizens. One can explore the right way to deal with this issue with reference to the way the dilemma was dealt with in the first century.

According to Greco-Roman ethics as posited by Cicero and Seneca, taking the vaccine by force would be a sin. That is to say, it would be forbidden for one country to steal vaccines from another on the grounds that it is committed to the security of its own citizens. Nevertheless, it is possible that Cicero, with his pragmatic Roman approach, would dare to say that when the interest of the state or of society is in the balance, it would be permitted to “steal” vaccines from another country, even if that would be likely to cause harm to the legitimate owner, who had duly purchased them. According to rabbinic texts on Jewish ethics, Rabbi Akiva would say that there is no obligation to worry about other countries until all the citizens of the state have been vaccinated, whereas according to Ben Petura, cooperation among people mandates sharing the global stock of vaccines with other countries (For elaboration regarding Judaism’s approach to vaccination and its stance that healing is a religious obligation, see: Rashi [32]).

## 4. Ethical Distribution of Vaccines to the General Population

Both the Sages of the Talmud and the Greek and Roman scholars addressed the situation in which two are traveling with just one water canteen, which is held by one of them. From this they attempted to deal with a fair distribution of the water. As the vaccines are not privately owned, but are rather the property of the nation, which is responsible for fair distribution among its citizens, the quandary in regard to the internal distribution of vaccines among citizens of a country can only be resolved by presenting an “upgraded” version of the initial dilemma. An appropriate analogy to the historical, philosophical dilemma would be if a third person were to meet up with two thirsty travelers who have no water. The third person has a canteen of water that will only suffice for a single individual, but he himself does not need it since he had just drunk enough water to reach a town. What should he do? According to Heyd [33], to share the water between them is an absurd and wasteful action since both will die of thirst (The position of Ben Petura in respect of the equal distribution of water that will lead to the death of both is an absurd, unethical solution in the case of a judge who is a third party, since he has no standing even with the argument of “do not see the death of your fellow” in the personal sense of saving his life at the expense of his companion. Owing to triage, a doctor may see people dying).

To give it to one of them when there is no ethical basis for his preference (e.g., if one is a child) is not a just solution. All that is left for the third person to do is to have the two of them draw lots. However, in any case, he must give the canteen to one of them and say, “You sort out the question of sharing with your companion, since I cannot judge to whom to give it.” However, it is virtually certain that the one who receives the water will not be fair as the third person intended but will follow in the way of Rabbi Akiva and drink all the water to save his own life. Thus, it can be said that a lottery should be the decision-making mechanism for the third party (“the distributor”), who has no personal interest in the distribution, which was not the case of “the two travelers.” 

According to Jewish tradition, the Ten Commandments were given to Moses at Mount Sinai some 3000 years ago in the presence of the entire Israelite nation (600,000 adult males). The complete Torah, written by Moses toward the end of his life, includes 613 commandments. 

Around these commandments and accompanying elaborations and clarifications (Written Law) there evolved an Oral Law comprising rabbinic discussions and arguments over the ensuing centuries that ultimately coalesced into the halakhah. In the second century of the Common Era, the period in which the first major codification of Jewish Law, the Mishnah, was written, rabbis set up a major center of scholarly religious learning to facilitate continuation of the halakhic tradition.

In the ensuing eighteen centuries, generations of religious leaders living in many countries around the world under the influence of various religiously oriented civilizations (primarily Catholic, Eastern Orthodox, Moslem, and Protestant) continued to broaden and further clarify and codify the Halakhah, which is a praxis-based code of law (i.e., legal principles are derived from specific problems and issues that arise in daily life, much like English Common Law). Thus, during the past 1500 years, tens of thousands of common-man “questions” and local rabbinical “answers” (in Hebrew: “shut”) have “clarified” the Halakhah, thereby developing what has come to be called “Responsa literature.” Every so often, owing to the unwieldiness of such a large corpus, major rabbinical commentators have taken it upon themselves to “codify” the law in some systematic and quasi-authoritative fashion [34]. 

Many rabbis have ruled that in light of the obligation to save life as established in Jewish law, (For example, Maimonides ruled in Laws of the Murderer and Preserving Life [35]: Anyone who can save a life and does not do so transgresses; “Do not stand by when your neighbor’s life is threatened” [16]) priority in medical treatment should be given to the one who is in a life-threatening situation (Such as Rabbi Shlomo Zalman Auerbach, in his book Minchat Shlomo [36]). That is, let us decide that it is preferable to treat the one likely to obtain the maximum benefit from the treatment [37]. Some of the rabbis who adopted this rule relied on two opinions: that of Rabbi Avraham Yishiyahu Karelitz (Eastern Europe and Israel, 1878–1953), a leading Ultra-Orthodox twentieth-century halachic arbiter and the one of Rabbi Isser Yehuda Unterman (1886–1976), a Chief Rabbi of Israel from 1964 to 1972. From the words of Rabbi Akiva, both of them concluded that if there is a resource that does not belong to either person who needs it, it should be given to the one who will derive the greatest benefit [38,39]. According to Rabbi Karelitz, one should give preference to one person rather than to extend the lives of both for a short time [38]. 

Rabbi Unterman also ruled the same way in the case of a doctor who was needed by many patients and could not get to all of them. As “the obligation... to give the patients enough care to heal them,” he should heal them for a long and normal life and not split himself among all of them such “that each receives only temporary relief” [39]. In that case the medicine was owned by a private person, and Rabbi Unterman added: “Since he is not ill now, one cannot say ‘your life takes precedence,’ since it is not a question of saving life.”) 

Rabbi Eliezer Waldenberg (1915–2006), a major twentieth-century halachic arbiter, a member of the Rabbinical High Court and the rabbi of the Shaarei Tzedek Hospital in Jerusalem, was known primarily for his Responsa in connection with medicine and halacha. In his book of Responsa *Tzitz Eliezer* [40], he described a case where there were two patients requiring the same medicine and the hospital did not have enough of a supply for both of them. Rabbi Waldenberg ruled that in this case it was not possible to follow the guidance of Rabbi Akiva that “your life takes precedence” since neither person was the owner of the medicine. Rather, he said, that it was Ben Petura’s opinion that should govern the situation and that the hospital should divide the medicine between them. Rabbi Waldenberg believed that this case was different from the two who were traveling in the desert, since in the medical world decisions are taken based on the judgment of the doctors in regard to both the severity of the patients’ conditions and the type of treatment required, so it was more reasonable to divide the medicine equally between them. 

What should the ruling be in a case of many patients and a single dose of medicine such as once occurred in Hadassah Hospital, Jerusalem? The hospital asked for advice from the sitting Chief Rabbi of Israel (1936–1959) Rabbi Yitzhak Isaac Herzog (1888–1959). A description of the case was presented by one of the doctors who was present during a discussion at a meeting of one of Israel’s parliament’s committees:

Many years ago, at the end of the Second World War, there occurred at Hadassah Hospital a most interesting case: the illness “bacterial meningitis” had spread, with a mortality rate of 100% until penicillin was discovered. When the drug arrived, which was only enough to treat one patient, there were 450 patients in the hospital. The dilemma arose as to who should be treated. The youngest of the child patients or an older person? Someone who held a senior position or someone unemployed? We contacted the then Chief Rabbi, Rabbi Herzog of blessed memory, to help us to make a decision. In the end it was decided that the doctor would treat the first patient he came upon in the department. How can we decide whose life is worth more? [40].

That is to say, as there was only enough of the drug for one patient, the entire supply would be used for the patient that the doctor saw first, as it was impossible to decide who had priority over whom. Rabbi Herzog’s approach was that everyone is equal and that no one has the authority to decide that the life of one individual is more important than the life of another. 

As noted above, it is worth discussing today’s global dilemma regarding the distribution of vaccines in light of the ancient rulings, principles, and norms of the various cultures. In regard to the distribution of vaccines today, opinions based on Jewish ethics are divided, even on moral terms. On the one hand, many believe that it is justified to distribute the available vaccine so as to provide long-term benefit; that is, to administer both doses to fewer people. This distribution is considered preferable to an equal distribution of single doses among twice the number of people on a first come, first served basis, all of whom will only be partially protected, as is the case with the British model. On the other hand, there is the minority opinion similar to that of Rabbi Waldenberg; that is, that in the medical world decisions are taken based on the judgment of the doctors, both in respect of the degree of danger to a patient and the type of treatment required, so in that case it was more reasonable to decide to divide the medicine equally between the patients.

## 5. Conclusions

The COVID-19 pandemic has forced the countries of the world to prioritize medical attention and prevention. The basis of democratic governance is a partnership among a country’s citizens. A country is obliged to provide equal treatment to all its citizens, especially in a case of a lifesaving activity. 

In his book *On Repentance*, Rabbi Yosef Dov Soloveitchik (1903–1993), a prominent twentieth-century religious leader in the United States, encapsulated the relationship between the individual and the public: “Never is the individual’s worth belittled measured against the whole community and never is the community undermined because of any individual or individuals. Each has its own position of strength” [41].

It is that relationship that is at the heart of the present essay. Finding the optimum balance between concern for the individual and for that of the collective requires careful thought. The foregoing pages offered viewpoints from Greco-Roman, Jewish, and Moslem philosophical debates in order to provide new perspectives regarding the way vaccines against COVID-19 should be distributed among countries and among the populations within each country.

This article presented the philosophical principles and not their implementation. Future researchers will have to analyze how the relevant decisions were taken in different places and the criteria that governed those decisions. 

## Data Availability

Not applicable.
